# Outstanding Reviewers for *Nanoscale Advances* in 2021

**DOI:** 10.1039/d2na90034c

**Published:** 2022-06-27

## Abstract

We would like to take this opportunity to highlight the Outstanding Reviewers for *Nanoscale Advances* in 2021, as selected by the editorial team for their significant contribution to the journal.
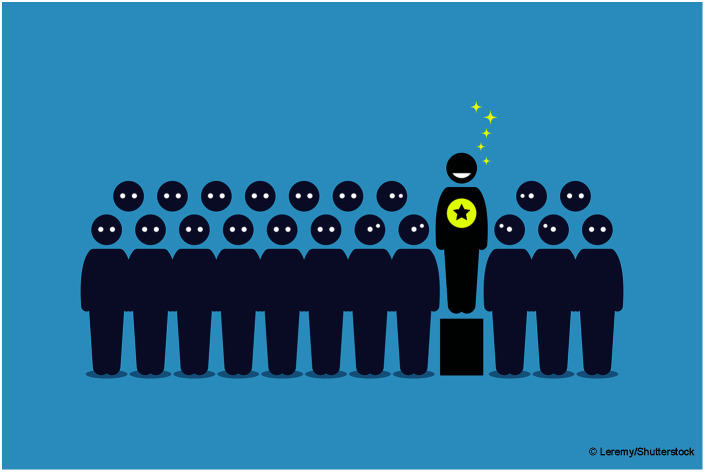

We would like to take this opportunity to thank all of *Nanoscale Advances*’ reviewers, and in particular highlight the Outstanding Reviewers for the journal in 2021. Selected by the editorial team and Editorial Board for their significant contribution to *Nanoscale Advances*, we announce our Outstanding Reviewers annually and each receives a certificate to give recognition for their contribution.

In recognition of the varied contributions of our reviewer community, our Outstanding Reviewers from 2021 have been chosen based on several different measures, including the number, timeliness and quality of the reports completed over the last 12 months. In addition to reviewers who provided a high number of quality reports, we are pleased to highlight reviewers who provided exceptionally thorough and detailed reports, reviewers who provided clear and insightful adjudicative reports and reviewers who made additional efforts to aid authors in improving their manuscripts.

 

“By now it is a good old tradition that *Nanoscale Advances* recognizes the Outstanding Reviewers. Key in terms of guaranteeing the quality and impact of *Nanoscale Advances* is the peer review process. As such, peer review depends not only on the excellence of the reviews but also on their timeliness. All of it comes on top of the many burdens that we face as active researchers. At the heart of the peer review process are carefully drafted reviews. They provide the valuable service that we owe to the scientific community in general, and to the readers of *Nanoscale Advances* in particular. I want to extend a big thank you to these Outstanding Reviewers and everyone else who has reviewed manuscripts for *Nanoscale Advances*.” – Professor Dirk Guldi, Editor-in-Chief

 

Dr Zibin Chen

The Hong Kong Polytechnic University

ORCID: 0000-0002-7144-1861

 

Dr Filippo Fabbri

Istituto Nanoscienze Consiglio Nazionale delle Ricerche

ORCID: 0000-0003-1142-0441

 

Dr Peng Huang

Shenzhen University

ORCID: 0000-0003-3651-7813

 

Dr Jun Li

University of Wisconsin–Madison

ORCID: 0000-0002-7498-6736

 

Dr Xin Tian

Soochow University (School of Radiation Medicine and Protection)

ORCID: 0000-0002-5631-8160

 

Dr Joshua Welsh

National Institutes of Health Institution

ORCID: 0000-0002-1097-9756

 

Dr Rui Yuan

PPG Industries Inc.

ORCID: 0000-0003-0063-0822

 

We would also like to thank the *Nanoscale Advances* Editorial Board and Advisory Board and the nanoscience community for their continued support of the journal, as authors, reviewers and readers.

 

Chunli Bai, Editor-in-Chief

Dirk Guldi, Editor-in-Chief

Jeremy Allen, Executive Editor

## Supplementary Material

